# Characterisation of metabolic burden in *Pseudomonas putida* reveals precursor limitation in heterologous lycopene production

**DOI:** 10.1186/s12934-026-03108-5

**Published:** 2026-09-02

**Authors:** Carina Meiners, Lucas Hermann, Mishela Stoja, Andreas Kremling, Katharina Pflüger-Grau

**Affiliations:** Technical University of Munich, Boltzmannstraße 15, 85748 Garching, Germany

**Keywords:** Metabolic burden, Precursor limitation, Terpenoid, Lycopene, *Pseudomonas putida*, Mevalonate pathway

## Abstract

**Background:**

The introduction of heterologous pathways into microbial hosts often imposes a metabolic burden on the cell, arising from three major physiological constraint layers: competition for gene expression resources, limited precursor availability and flux distribution, and insufficient energy and redox supply. Although *Pseudomonas putida* KT2440 is considered a robust and metabolically versatile production host, it remains unclear which of these constraint layers primarily limits heterologous terpenoid production in this organism. Here, lycopene biosynthesis was used as a model system to systematically dissect these three potential sources of metabolic burden.

**Results:**

A capacity-monitoring system revealed no clear reduction in transcriptional or translational capacity across the tested strains and cultivation conditions, indicating that general gene expression capacity was not the primary limiting factor. Instead, lycopene production depended strongly on promoter architecture and plasmid backbone, showing that regulatory design shaped pathway performance. Enhancing precursor supply by introducing a heterologous mevalonate (MVA) pathway substantially increased product titres, identifying precursor availability from the native MEP pathway as the dominant bottleneck. This conclusion was independently supported by exogenous mevalonate supplementation, which further increased lycopene accumulation but also revealed saturation at higher concentrations, suggesting that downstream pathway balance or enzyme capacity became limiting once precursor supply was relieved. Under controlled bioreactor conditions, lycopene titres increased from approximately 1 mg/L to nearly 25 mg/L, indicating that process conditions further modulate production performance, suggesting an additional contribution of process-dependent energy and redox constraints.

**Conclusion:**

Metabolic burden during heterologous lycopene production in *P. putida* is governed primarily by precursor availability rather than by limitations in general gene expression capacity. Regulatory properties of the vector system strongly influence pathway performance, while controlled cultivation conditions can further improve production by alleviating additional process-dependent constraints. Together, these findings provide a systematic framework for distinguishing constraint layers and guiding the optimisation of heterologous terpenoid production systems.

**Graphical Abstract:**

**Figure:**
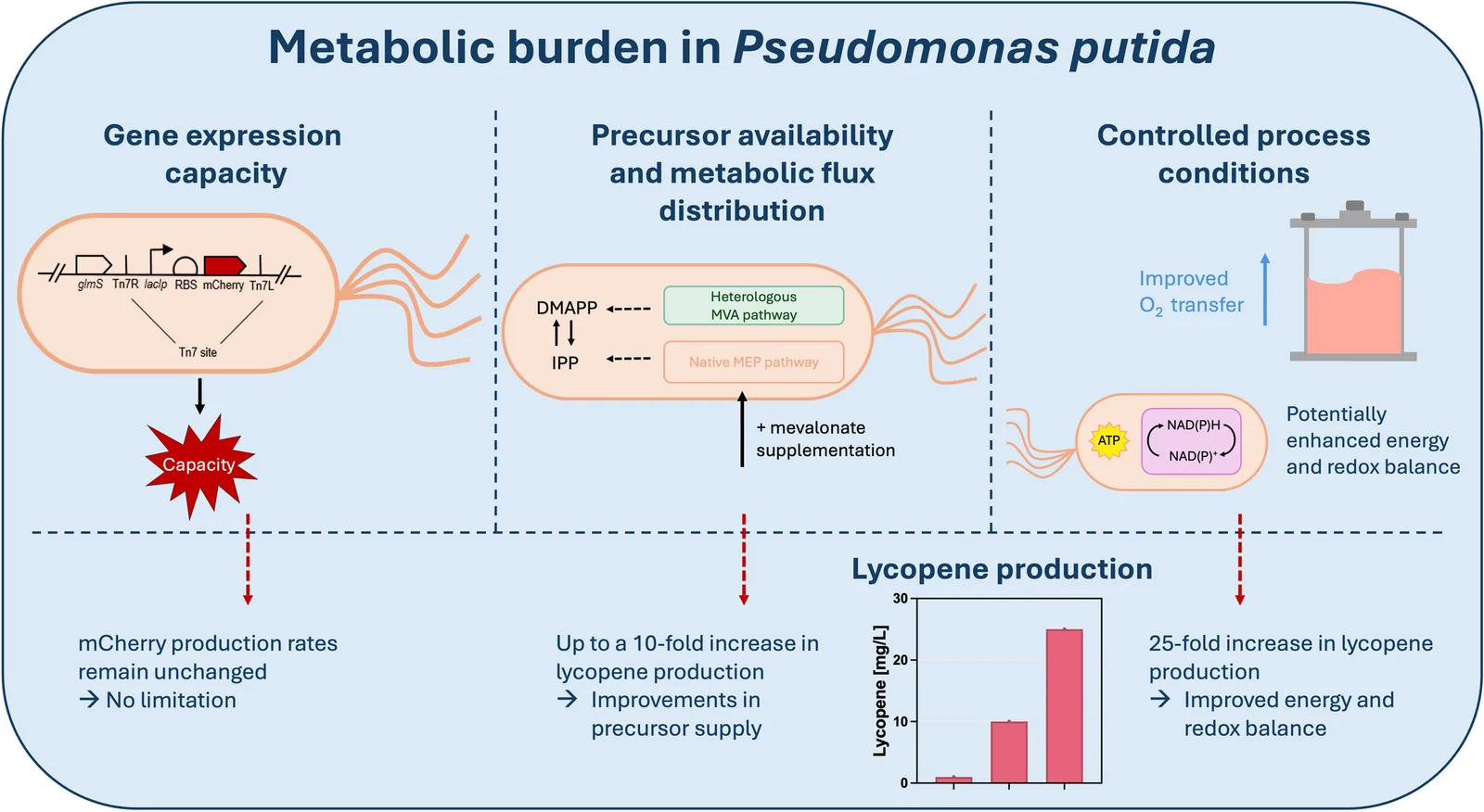

**Supplementary Information:**

The online version contains supplementary material available at https://doi.org/10.1186/s12934-026-03108-5.

## Background

**Fig. 1:**
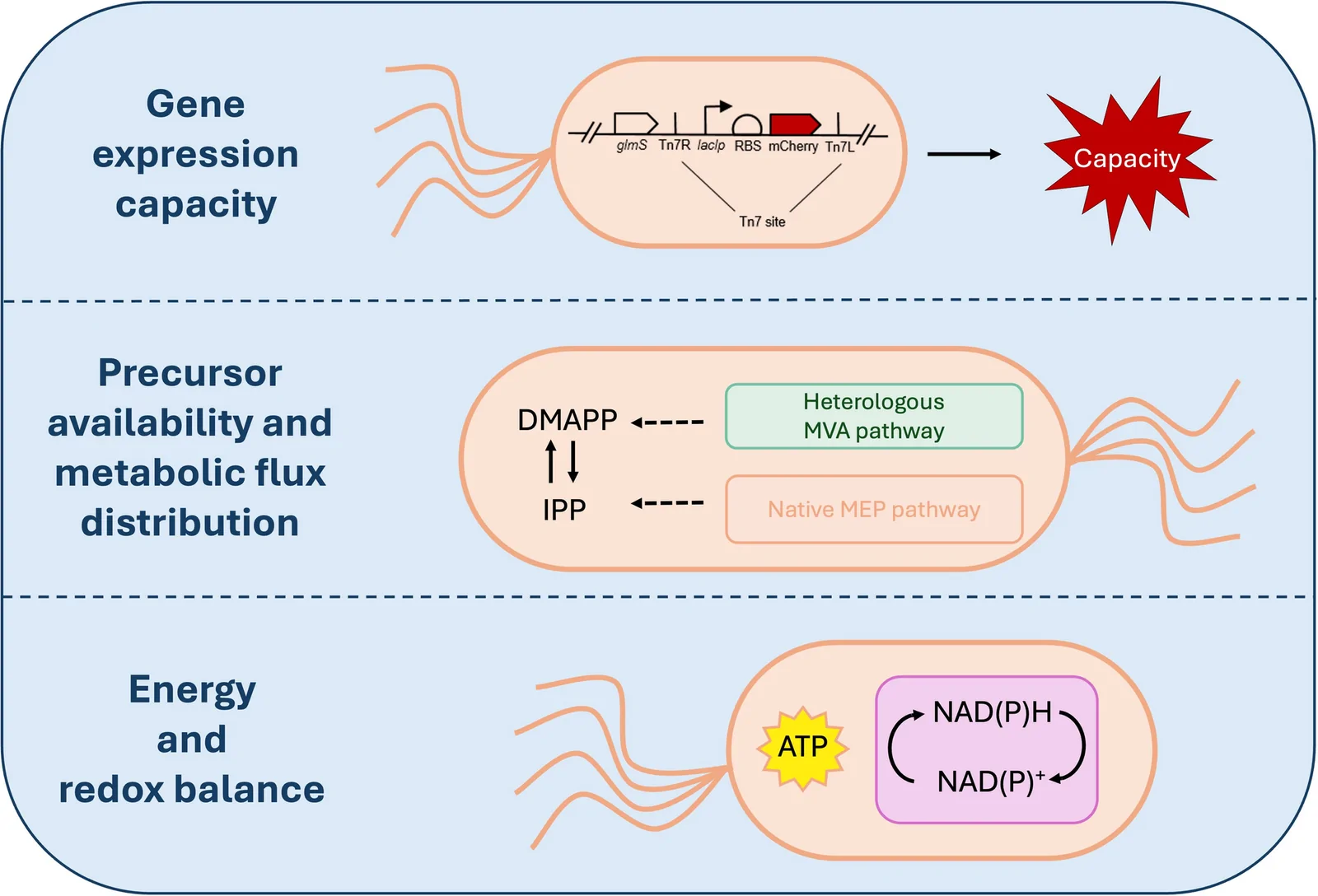
Key determinants of metabolic burden during heterologous pathway expression. Metabolic burden arises from limitations in gene expression capacity, including transcriptional and translational resources, from precursor availability and metabolic flux distribution, represented by the native MEP pathway and the heterologous MVA pathways supplying the precursors IPP and DMAPP; and from energy and redox balance, determined by the intracellular availability of ATP and NAD(P)H. These interconnected physiological processes collectively determine the performance of heterologous pathways and cellular fitness. IPP: Isopentenyl pyrophosphate; DMAPP: dimethylallyl pyrophosphate; NAD(P): Nicotinamide adenine dinucleotide (phosphate)

The introduction of heterologous metabolic pathways into bacterial hosts frequently affects the native physiological balance of the cell, resulting in a metabolic burden negatively affecting growth rates and production performance. At the cellular level, this burden arises from the combined action of three interconnected physiological processes (Fig. 1): (i) gene expression capacity, (ii) precursor availability and metabolic flux distribution, and (iii) energy and redox balance.

Gene expression capacity can be affected by competition between native and heterologous systems for the transcriptional and translational machinery. In parallel, engineered pathways can imbalance intracellular metabolite distribution by drawing intermediates away from native metabolism, thereby limiting precursor availability and reducing pathway efficiency. Furthermore, heterologous production often increases the cellular demand for ATP and reducing equivalents such as NAD(P)H, which can become particularly critical in highly reductive biosynthetic pathways [1–4]. Together, these factors can substantially impair cellular fitness, metabolic stability, and overall production capacity.

The soil bacterium *Pseudomonas putida* KT2440 is a robust and metabolically versatile Gram-negative organism with high redox capacity, broad substrate utilisation, and pronounced tolerance toward environmental and metabolic stress [5–8]. These properties have made *P. putida* a promising microbial chassis for the biosynthesis of value-added compounds, including redox-demanding terpenoids [9–13].

Among terpenoid products, carotenoids and particularly lycopene, have gained considerable attention as model products for microbial production systems. Lycopene, a C_40_-carotenoid, is a high-value red pigment with applications in the food, pharmaceutical, and cosmetic industries due to its antioxidant, anti-inflammatory, and anticancer properties [14]. It is traditionally obtained from plant sources such as tomatoes, papayas, or watermelons; however, extraction processes are energy-intensive, yield low product levels, and raise environmental concerns [15, 16]. Consequently, microbial production through metabolic engineering has emerged as an attractive alternative, with established platforms such as *Escherichia coli*, *Saccharomyces cerevisiae*,* Yarrowia lipolytica*, and *Pichia pastoris*, with some highly optimised systems reaching multi-gram-per-litre titres, enabling efficient, scalable terpenoid biosynthesis [17–24]. Nevertheless, efficient microbial synthesis of terpenoids is frequently limited by the availability of the universal C_5_-precursors isopentenyl diphosphate (IPP) and dimethylallyl diphosphate (DMAPP), which often fail to sustain the high metabolic flux required for isoprenoid formation [4, 25]. Such precursor limitations can create metabolic bottlenecks and intensify the metabolic burden associated with precursor supply and flux distribution, particularly when heterologous enzymes compete for the same intracellular precursor pools.

*P. putida* natively relies exclusively on the methylerythritol phosphate (MEP) pathway for IPP and DMAPP synthesis. Although energetically efficient, the MEP pathway is tightly regulated and typically cannot supply sufficient precursor flux to support robust terpenoid production [26]. A widely applied strategy to overcome this limitation is the additional introduction of the mevalonate (MVA) pathway. Supplementing the native isoprenoid metabolism with the MVA pathway can increase intracellular precursor availability, improve carbon allocation toward terpenoid biosynthesis, and substantially enhance final product titres [27–30]. In contrast to the MEP pathway, which is predominantly found in bacteria, the MVA pathway is naturally used by archaea and most eukaryotes, including fungi and animals [31].

In this study, the impact of heterologous pathway implementation on metabolic burden in *P. putida* was systematically investigated using lycopene production as a model system. To examine the three physiological processes contributing to metabolic burden, different experimental approaches were employed. To assess the contribution of gene expression capacity, a *P. putida* reporter strain carrying a genomically integrated fluorescent reporter under the control of a constitutive promoter was used [32]. The fluorescence can therefore be used as a proxy for general gene expression capacity. In addition, lycopene biosynthesis was implemented using two distinct promoter systems to evaluate the influence of vector architecture on pathway performance. The role of precursor supply and flux distribution was investigated by enhancing isoprenoid precursor supply through the introduction of a heterologous MVA pathway as well as by supplementation with its key intermediate, mevalonate. Finally, cultivation in a controlled bioreactor environment was used to evaluate the influence of improved process conditions on lycopene production, potentially reflecting changes in energy and redox metabolism. This experimental framework enabled systematic differentiation of limitations arising from gene expression capacity, precursor supply, and the availability of energy and redox cofactors.

## Methods

### Strains and culture conditions

All *Escherichia coli* and *Pseudomonas putida* KT2440 *attTn7::lacIp-mCherry* (hereafter referred to as *P. putida* CAP [32]) strains were cultured at 37 °C–30 °C, respectively, with shaking at 180 rpm. For growth experiments, the following preculture procedure was used: in the initial preculture step, the strains were grown on LB agar plates (10 g/L tryptone; 5 g/L yeast extract, 10 g/L NaCl) supplemented with the respective antibiotic for 24 h: kanamycin (50 µg/mL) for strains harbouring pSEVA2312-based plasmids or streptomycin (100 µg/mL) for strains carrying pSEVA438 derivatives. Next, a single colony was picked to inoculate LB liquid medium containing the corresponding antibiotic. After another 24 h of incubation, this LB preculture was used to inoculate M9 medium [33] to an initial optical density at 600 nm (OD_600_) of 0.1. The medium was supplemented with the appropriate antibiotic and glucose as the carbon source, 3 g/L for shake flask experiments and 15 g/L for the bioreactor experiment. The following day, these cultures were used to inoculate the main cultures. For the experiments with MVA supplementation, this compound was added to the M9 medium at the start of the cultivation to the respective final concentrations. Expression of genes encoded on pSEVA438-derived plasmids under the control of the XylS/P_*m*_ promoter was induced with 1 mM 3-methylbenzoate (3-MB). Genes encoded on pSEVA2312-derivatives under the control of the CprK1/P_*DB3*_ promoter were induced with 1 mM 3-chloro-4-hydroxyphenylacetate (CHPA).

### Growth monitoring and mCherry quantification

The optical density of cultures was measured at 600 nm (OD_600_). The OD of precultures and bioreactor samples was measured using a BioSpectrometer^®^ (Eppendorf, Germany) in 1.5-mL cuvettes, whereas the OD of growth experiments in shake-flask was monitored using an Infinite^®^ M200 Pro microplate reader (Tecan, Switzerland) with 96-well plates and a sample volume of 200 µL per well. To ensure comparability between measurement systems, OD_600_ values obtained from the microplate reader were corrected using a conversion factor. A path length correction factor of 1.75 was applied to all OD_600_ measurements obtained from the microplate reader to ensure comparability with standard 1 cm cuvette measurements. This factor was determined based on the difference in optical path length between the microplate wells and the cuvette.

The genomically integrated red fluorescent protein mCherry was used as a reporter to monitor cellular expression capacity. Fluorescence measurements were performed using the microplate reader (Infinite^®^ M200 Pro, Tecan, Switzerland). mCherry fluorescence was recorded at an excitation wavelength of 580 nm and an emission wavelength of 615 nm. To determine the mCherry production rate, fluorescence data were fit to a linear regression over the same time window used to calculate the specific growth rate. Growth rates were determined from data points collected only during the exponential phase, when growth is constant in batch culture. This phase was identified by linear regression of ln(OD_600_) over time, and the selected time window corresponded to the interval with the highest coefficient of determination (R²) across the full growth curve. The exact intervals are listed in Table S1 in the supplementary information.

### Plasmids

To construct the plasmids pSEVA438-Lyc and pSEVA2312-Lyc, the lycopene operon, consisting of the *crtE*, *crtB*, and *crtI* genes from *Pantoea ananatis*, was amplified from the plasmid pTn7-Lyc [34] using Phusion High-Fidelity DNA Polymerase (New England Biolabs, UK) and primers containing 15–25 bp overlaps to the corresponding vector backbones (see Table 1 for all oligonucleotides used in this work). The vector backbones (pSEVA438 and pSEVA2312) were amplified using primers that provided complementary overlaps to the lycopene operon fragment. The resulting PCR fragments were assembled using the NEBuilder^®^ HiFi DNA Assembly (New England Biolabs, USA).

For the construction of the plasmids pSEVA438-Lyc-MVA and pSEVA2312-Lyc-MVA, the same strategy was applied, except that the previously constructed plasmids pSEVA438-Lyc and pSEVA2312-Lyc served as templates for vector amplification. The MVA operon, consisting of six genes (*hmgs*, *idi*, *hmgr*, *mvk*, *mvd*, *pmvk*) from *Myxococcus xanthus*, was amplified from the plasmid pMiS1-ges-MVA [35] and assembled downstream of the respective promoter systems in the vector backbones using the NEBuilder^®^ HiFi DNA Assembly Kit (New England Biolabs, USA). All genes were transcribed from the plasmid-specific expression system, retaining their native ribosome-binding sites (RBSs).

The constructed plasmids were first chemically transformed into *Escherichia coli* DH5α for propagation and verification [36] and were subsequently introduced into *P. putida* CAP by electroporation [37]. Plasmid integrity and sequence accuracy were confirmed by whole-plasmid sequencing (Azenta Life Science, USA).

**Table 1:** Oligonucleotides used in this work, sorted according to their application

Oligonucleotide	Sequence (5’-3’)	
Construction of pSEVA438-Lyc		
Lyc-operon_438_fwd		TGCAAGCTTGAATTCGAGCTCGGTACCGCAC
Lyc-operon_438_rev		AGACTAGTCGCTAGAGCGGGCGCTGCCA
pSEVA438_fwd		CCCGCTCTAGCGACTAGTCTTGGACTCCTG
pSEVA438_rev		AGCTCGAATTCAAGCTTGCATGCCTGCAG
Construction of pSEVA2312-Lyc		
Lyc-operon_2312_fwd		CCGGGGATCCAATTCGAGCTCGGTACCGCAC
Lyc-operon_2312_rev		CGACTCTAGACTAGAGCGGGCGCTGCCA
pSEVA2312_fwd		CCCGCTCTAGTCTAGAGTCGACCTGCAGG
pSEVA2312_rev		AGCTCGAATTGGATCCCCGGGTACCGAG
Construction of pSEVA438-Lyc-MVA		
pSEVA438-Lyc_fwd		TGAAAGCTTATAGTCTTGGACTCCTGTTGATAGATCCAG TAATG
pSEVA438-Lyc_rev		AGCGCCCGCTCTAGCGACCCTCGAGTGT
MVA_438_fwd		CTCTAGCGACCCTCGAGTGTACAGGATC
MVA_438_rev		CGCTGAGCTGAAAGCTTATAGTCTTGGA
Construction of pSEVA2312-Lyc-MVA		
pSEVA2312-Lyc_fwd		GCTGAGCTGACAAGCTTGCGGCCGCGTC
pSEVA2312-Lyc_rev		CTAGAGTCGACCTGCAGGCATGCCTCGAGTGT
MVA_2312_fwd		TGCAGGCATGCCTCGAGTGTACAGGATCCAGGAG
MVA_2312_rev		GTGCGCGCGCTGAGCTGACAAGCTTGCG

### Extraction and quantification of lycopene

Expression was induced with 1 mM 3-MB for the pSEVA438-based systems and 1 mM CHPA for the pSEVA2312-based systems after 4 h of cultivation in shake flasks and after 5 h in the bioreactor. Samples (5 mL) were collected at various time points and frozen immediately. The final sample was taken after 24 h. For lycopene extraction [38], samples were thawed and centrifuged at 4,000 × g for 15 min at 4 °C. The supernatant was discarded, and the resulting cell pellet was resuspended in 1 mL of acetone. The suspensions were incubated in the dark at 55 °C for 15 min to prevent lycopene degradation. Subsequently, the samples were centrifuged again at 12,000 × g for 10 min, and the absorbance of the supernatant was measured at 472 nm. Lycopene concentrations were determined using a standard curve prepared from lycopene standards dissolved in acetone at concentrations between 0.001 and 0.01 g/L.

### Bioreactor cultivation

Lycopene production in the bioreactor was performed in a batch process in a 3.6 L stirred-tank bioreactor (Labfors 5, Infors GmbH, Bottmingen, Switzerland). The reactor was equipped with two six-bladed Rushton impellers and three baffles. Prior to process start, 1 L of sterile M9 medium containing 15 g/L glucose and 50 mg/L kanamycin was added to the reactor. Throughout the process, a temperature of 30 °C was maintained. The pH was monitored using a two-point-calibrated pH probe (EasyFerm Plus PHI Arc 325, Hamilton Bonaduz AG, Bonaduz, Switzerland) and maintained at pH 7 by the addition of 42% phosphoric acid and 25% ammonia. The dissolved oxygen concentration (pO₂) was measured using a single-point calibrated pO₂ probe (VisiFerm DO Arc 325 H0, Hamilton Bonaduz AG, Bonaduz, Switzerland). The initial stirred speed was set to 500 rpm with an aeration rate of 1 L min⁻¹, and the stirrer speed was increased as necessary to maintain a pO₂ above 40%. To prevent foam formation, a small amount of antifoam solution (AF204, Sigma-Aldrich, Taufkirchen, Germany) was added right after inoculation. The concentrations of oxygen (O₂) and carbon dioxide (CO₂) in the off-gas were measured online using a gas analyser (BlueInOne Ferm, BlueSens, Herten, Germany). The bioreactor was inoculated with an initial OD_600_ of 0.1, and lycopene production was induced 5 h post-inoculation by the addition of 1 mM CHPA.

## Results

### Engineering of capacity-reporting *P. putida* strains for stepwise optimisation of lycopene production

In this work, the previously established capacity-monitoring strain *P. putida* CAP was used to evaluate the impact of the heterologous lycopene production pathway on cellular gene expression capacity. This strain carries a genomically encoded constitutively expressed mCherry reporter cassette, whose fluorescence signal serves as an indirect proxy for cellular expression capacity. Accordingly, reductions in mCherry fluorescence indicate increased competition for transcriptional and translational resources caused by heterologous gene expression [32, 39].

**Fig. 2:**
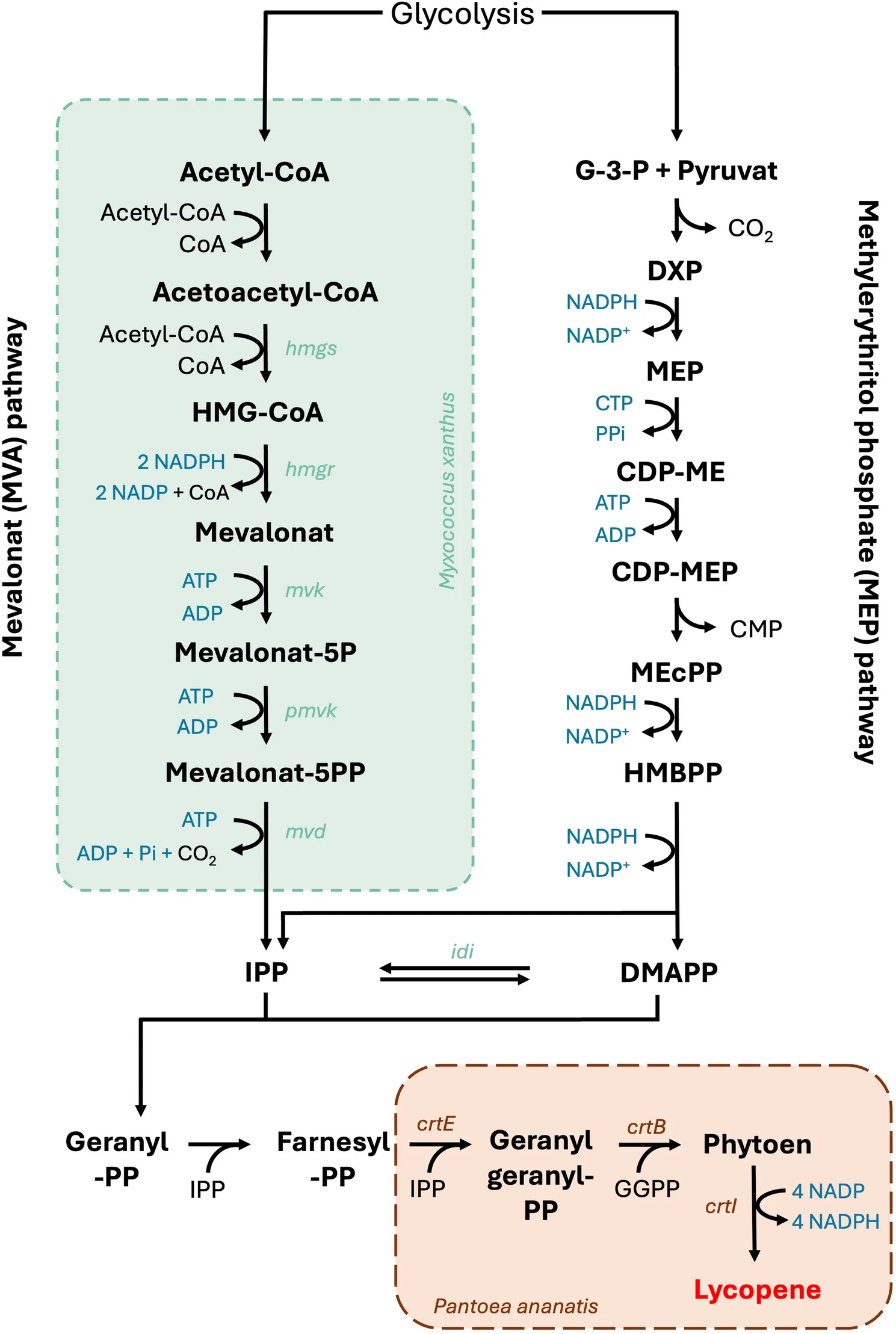
Heterologous pathway for lycopene production in *P. putida* CAP (pSEVA2312-Lyc-MVA) and *P. putida* CAP (pSEVA438-Lyc-MVA). Genes of the MVA pathway derived from *Myxococcus xanthus* are highlighted in green, while genes of the lycopene pathway derived from *Pantoea ananatis* are shown in red. Reactions intrinsic to *P. putida* are shown without background colouring. CoA: Coenzyme A; HMG-CoA: 3-hydroxy-3-methylglutaryl coenzyme A; G-3-P: D-Glyceraldehyde 3-phosphate; DXP: 1-deoxy-D-xylulose 5-phosphate; MEP: methylerythritol phosphate; CDP-ME: 2 C-methyl-D-erythritol 4-phosphate; CDP-MEP: 4-diphosphocytidyl-2 C-methyl-D-erythritol; MEcPP: 4-diphosphocytidyl-2 C-methyl-D-erythritol 2-phosphate; HMBPP: 2 C-methyl-D-erythritol 2,4-cyclodiphosphate; IPP: Isopentenyl pyrophosphate; DMAPP: dimethylallyl pyrophosphate; GGPP: geranylgeranyl pyrophosphate; NADPH: nicotinamide adenine dinucleotide phosphate; CTP: cytidine triphosphate; CMP: cytidine monophosphate

To establish heterologous terpenoid production in *P. putida* CAP, the lycopene biosynthesis operon (*crtE*, *crtB*, *crtI*) from *Pantoea ananatis* was introduced using two distinct expression vectors, pSEVA438 and pSEVA2312, which differ primarily in promoter architectures and induction strategy [40]. The two systems were selected to compare a widely established, host-native expression system with an alternative heterologous system designed to provide tighter transcriptional control. The pSEVA438 vector carries the XylS/P_*m*_ system, which originates from the *P. putida* TOL plasmid pWW0 and is commonly used for heterologous gene expression in this host. In contrast, pSEVA2312 employs the heterologous CprK1/P_*DB3*_ system and was included to examine whether more stringent transcriptional regulation and reduced basal expression could improve pathway performance. This design enabled a comparative evaluation of how the vector architecture and expression control influence lycopene production and cellular expression capacity. The enzymes encoded by the lycopene biosynthesis operon convert the methylerythritol phosphate-derived isoprenoid precursors IPP and DMAPP into the C_40_ carotenoid lycopene through three enzymatic steps (Fig. 2). First, the geranylgeranyl diphosphate synthase CrtE synthesises geranylgeranyl diphosphate (GGPP), the specific precursor for carotenoid formation. CrtB, a phytoene synthase, then condenses two GGPP molecules to phytoene, and the phytoene desaturase CrtI subsequently introduces a series of desaturation reactions that yield all-trans-lycopene. Because these reactions require substantial carbon flux and redox cofactors, the pathway most likely imposes a metabolic burden on the host. The resulting strains therefore provide a model system for investigating how heterologous lycopene biosynthesis affects cellular resource allocation and competes with endogenous cellular processes.

### Promoter-dependent differences in lycopene production in *P. putida* CAP

To investigate the influence of the two expression systems on heterologous pathway performance, the strains *P. putida* CAP (pSEVA438-Lyc) and *P. putida* CAP (pSEVA2312-Lyc) were compared with and without the addition of the respective inducer. Growth, lycopene production, and cellular gene expression capacity were monitored to evaluate the impact of promoter architecture and induction strategy on pathway expression and metabolic burden.

**Fig. 3:**
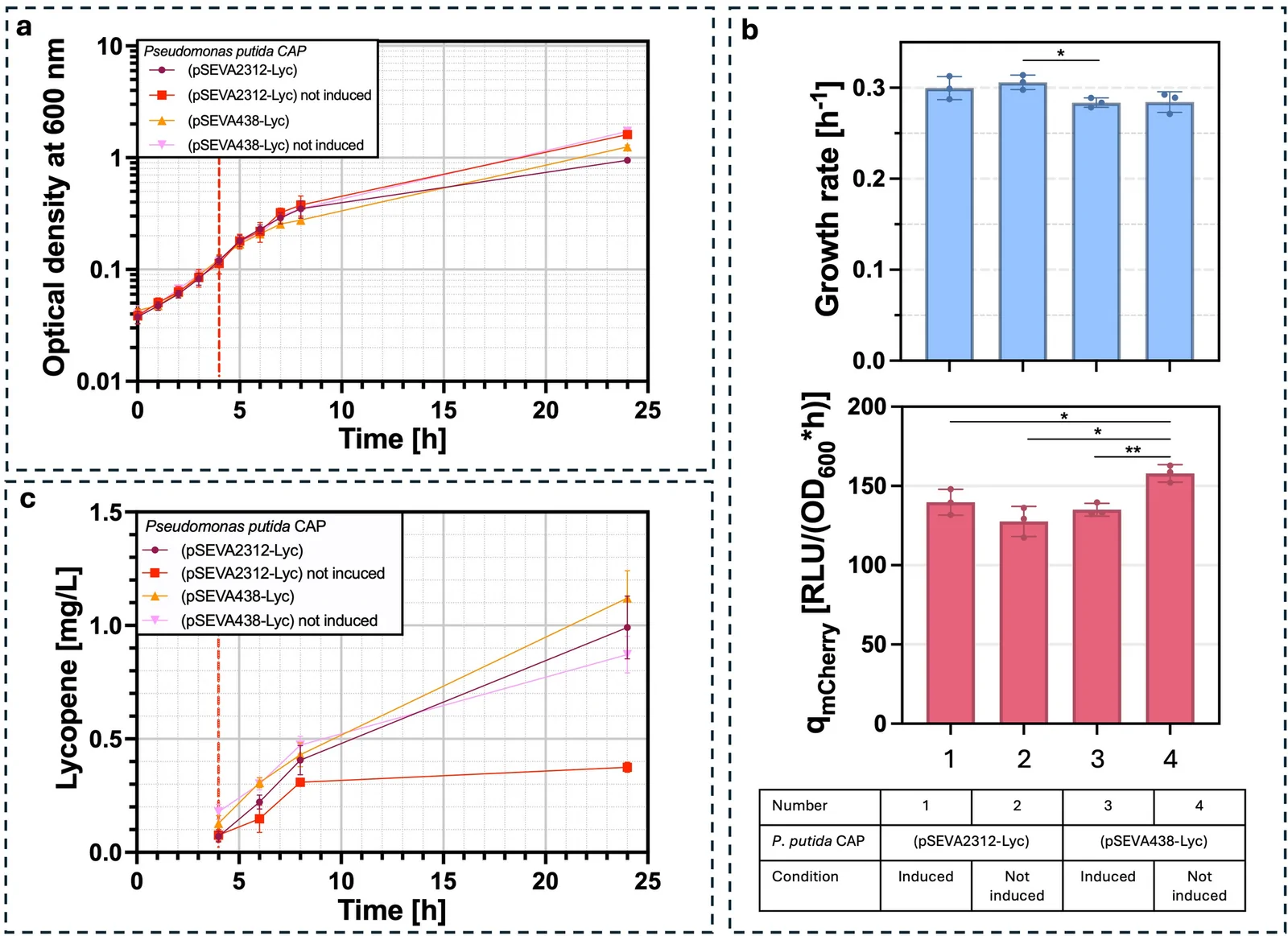
Growth (**a**), growth rate and capacity estimated from mCherry fluorescence rates (**b**), and lycopene production (**c**) of *P. putida* CAP carrying pSEVA438-Lyc or pSEVA2312-Lyc. Cultivation conditions: Triplicates in M9 mineral medium with glucose (3 g/L), shake flasks, 30 °C, induction with 1 mM 3-MB or CHPA, respectively, after 4 h (dotted line), shown is the mean and the standard deviation of the replicates (*n* = 3). The asterisks represent a statistical difference (*p* < 0.05 (*) and *p* < 0.01 (**) for an unpaired two-tailed t-test)

Growth profiles were highly similar across all cultures, with no observable differences between promoter systems or between the presence or absence of the inducer (Fig. 3a). All strains exhibited comparable growth rates of approximately 0.3 h^− 1^. The gene expression capacity, approximated from the mCherry fluorescence rates, was similar comparing inducing and non-inducing conditions in *P. putida* carrying the pSEVA2312-based construct, whereas for *P. putida* (pSEVA438-Lyc), a significant difference was observed (induced:135 ± 4.07 RLU/(OD_600_*h) vs. not induced: 157.92 ± 5.45 RLU/(OD_600_*h) (Fig. 3b).

Lycopene was produced by all strains, including the non-induced controls (Fig. 3c). The strain carrying the plasmid with the XylS/P_*m*_ promoter showed comparable lycopene titres under inducing and non-inducing conditions, reaching 1.12 ± 0.12 mg/L and 0.87 ± 0.08 mg/L, respectively. In contrast, *P. putida* CAP (pSEVA2312-Lyc) carrying the plasmid with the CprK1/P_*DB3*_ promoter accumulated considerably more lycopene when expression was induced, reaching a titre of 0.99 ± 0.14 mg/L, compared to 0.37 ± 0.02 mg/L under non-inducing conditions.

Overall, the CprK1/P_*DB3*_-based construct exhibited lower basal expression and showed a clear response to inducer addition, while the XylS/P_*m*_-based construct showed an inducer-independent lycopene production. These results indicate that differences in lycopene production are better explained by regulatory properties of the expression system than by a global limitation in cellular expression capacity.

### Enhancing isoprenoid precursor supply through heterologous expression of the MVA pathway

Following the observation that lycopene pathway expression did not noticeably affect growth or cellular capacity in *P. putida* CAP, we next examined whether precursor supply, rather than the general expression burden, constrained product formation. To increase the intracellular supply of isoprenoid precursors IPP and DMAPP, six genes encoding the MVA pathway from *M. xanthus* (*hmgs*, *idi*, *hmgr*, *mvk*, *mvd*, *pmvk*) were cloned downstream of the lycopene operon into pSEVA438-Lyc and pSEVA2312-Lyc. This established a heterologous MVA pathway in *P. putida* CAP in addition to the native MEP pathway. The MVA pathway converts acetyl-CoA into the universal isoprenoid precursors IPP and DMAPP. It is initiated by the condensation of acetyl-CoA to form 3-hydroxy-3-methylglutaryl coenzyme A (HMG-CoA), which is subsequently reduced to mevalonate. Mevalonate is then phosphorylated and decarboxylated to yield IPP, which can be further isomerised to DMAPP. The complete pathway is illustrated in detail in Fig. 2.

The resulting *P. putida* CAP strains carrying the plasmids pSEVA438-Lyc-MVA or pSEVA2312-Lyc-MVA were analysed after 24 h of cultivation in M9 minimal medium, by monitoring growth, capacity, and lycopene production.

**Fig. 4:**
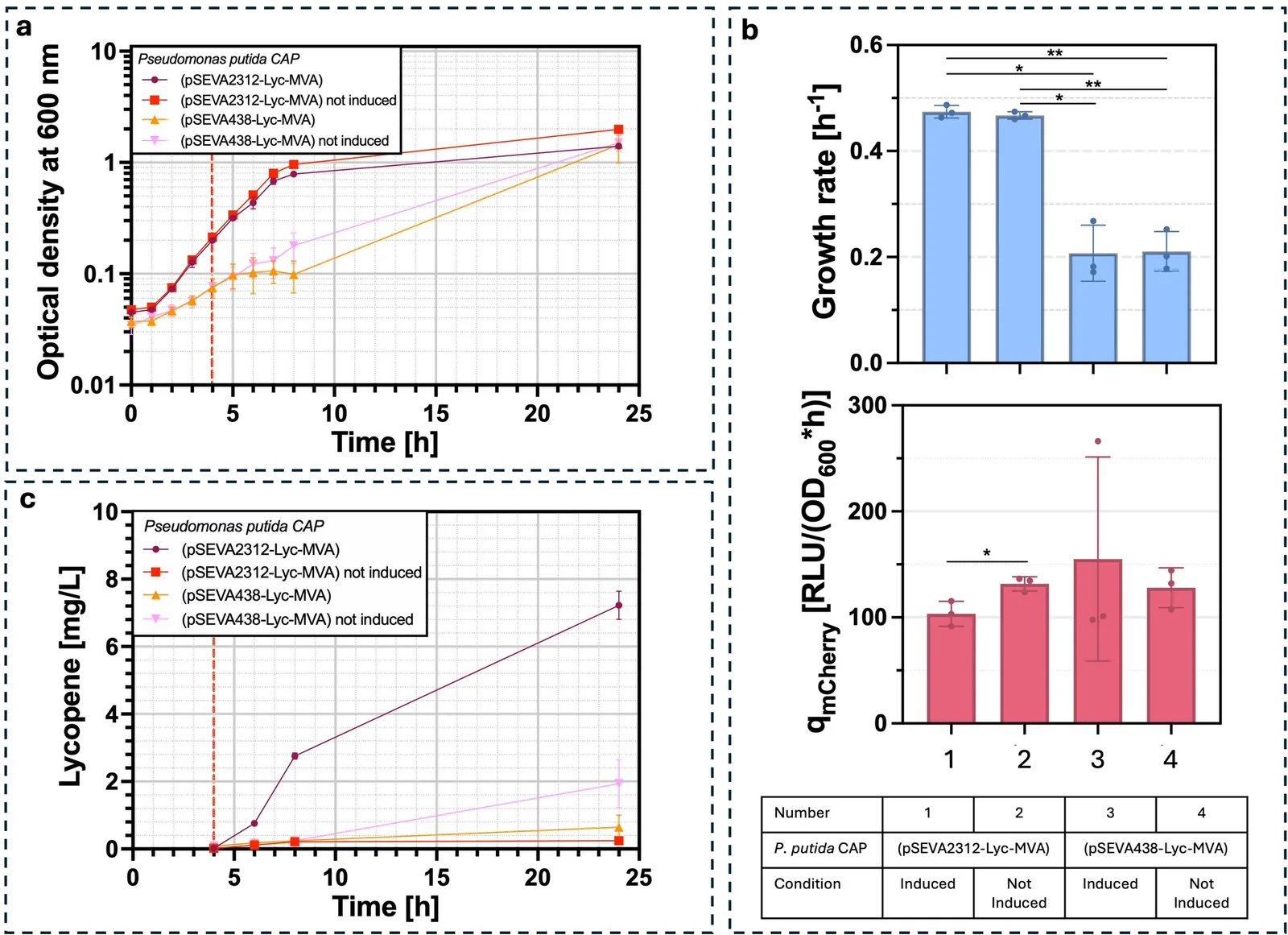
Growth (**a**), growth rate and capacity estimated from mCherry fluorescence rates (**b**), and lycopene production (**c**) of *P. putida* CAP carrying pSEVA438-Lyc-MVA or pSEVA2312-Lyc-MVA. Cultivation conditions: Triplicates in M9 mineral medium with glucose (3 g/L), shake flasks, 30 °C, induction with 1 mM 3-MB or CHPA, respectively, after 4 h (dotted line). The exact time points used to calculate the growth rate and the mCherry production rate are provided in the supplements. The asterisks represent a statistical difference (*p* < 0.05 (*) and *p* < 0.01 (**) for an unpaired two-tailed t-test)

Depending on the promoter system, a difference in growth behaviour was observed, with *P. putida* CAP (pSEVA438-Lyc-MVA) exhibiting markedly slower growth than *P. putida* CAP (pSEVA2312-Lyc-MVA) (Fig. 4a). This difference was also reflected in the specific growth rates (Fig. 4b). The strain carrying pSEVA438-Lyc-MVA achieved similar growth rates of around 0.21 h^− 1^ in induced and non-induced conditions, whereas the strain carrying pSEVA2312-Lyc-MVA reached substantially higher growth rates of 0.47 h^− 1^ in both conditions.

Despite these differences observed during exponential growth, all strains reached comparable final optical densities of around OD_600_ ≈ 1.5.

*P. putida* (pSEVA2312-Lyc-MVA) showed a significantly higher mCherry production rate in non-inducing conditions compared to inducing conditions. However, no significant differences in the mCherry production rate were detected comparing the different promoter systems, despite the pronounced differences in the growth rates of the respective strains (Fig. 4b). Lycopene accumulation was strongly influenced by both the choice of the expression system and the induction strategy (inducing vs. non-inducing conditions) (Fig. 4c). Among all tested conditions, *P. putida* CAP (pSEVA2312-Lyc-MVA) exhibited the highest lycopene titre under inducing conditions, reaching 7.23 ± 0.42 mg/L after 24 h. In the absence of the inducer, lycopene production decreased to 0.25 ± 0.06 mg/L. In contrast, induction of the XylS/P_*m*_-controlled genes from pSEVA438-Lyc-MVA resulted in lower lycopene accumulation (0.65 ± 0.35 mg/L) than in cultivations without inducer (1.94 ± 0.72 mg/L).

While vector architecture strongly shaped the magnitude of the response, the marked increase in lycopene titre upon introduction of the MVA pathway identifies precursor supply as the major bottleneck in the system.

### Mevalonate supplementation enhances lycopene production in *P. putida* CAP

**Fig. 5:**
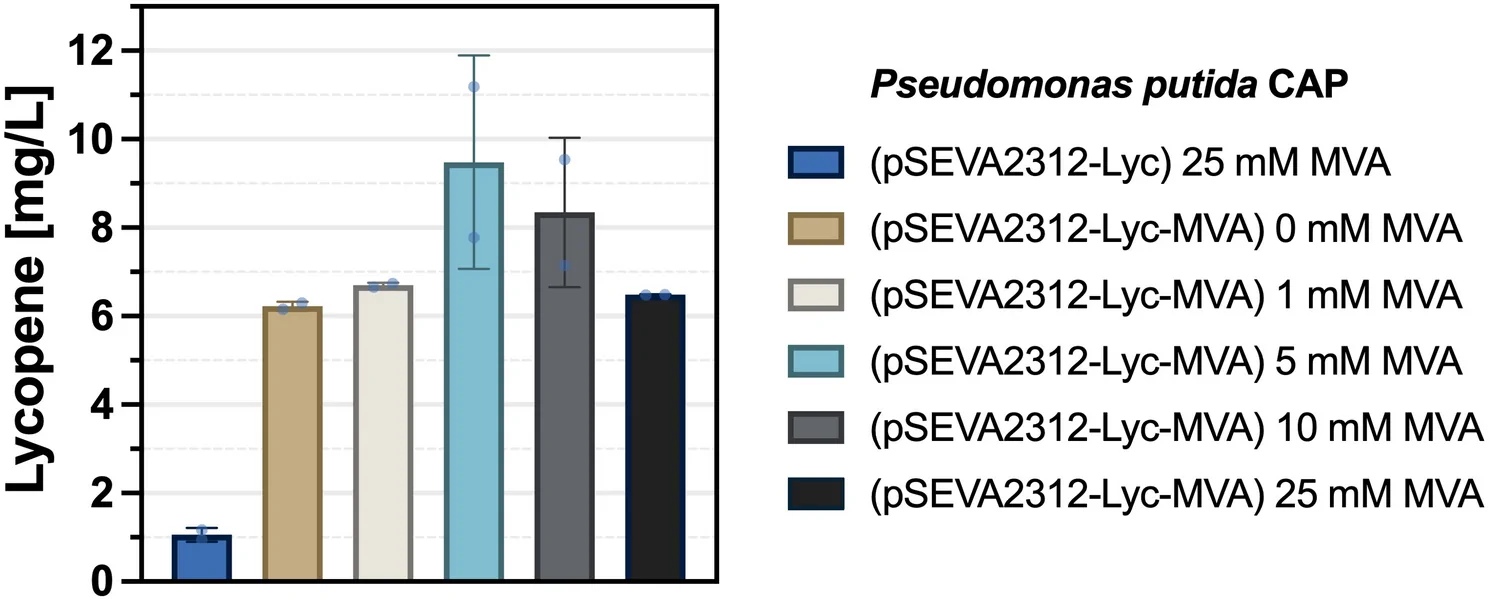
Lycopene content in *P. putida* CAP (pSEVA2312-Lyc) and *P. putida* CAP (pSEVA2312-Lyc-MVA) after 24 h of cultivation supplemented with different concentrations of mevalonate. Shown is the amount of lycopene extracted from cultures after 24 h. Shown from left to right are *P. putida* CAP (pSEVA2312-Lyc) supplemented with 25 mM MVA (dark blue), *P. putida* CAP (pSEVA2312-Lyc-MVA) supplemented with 0 mM MVA (sand-coloured), 1 mM MVA (beige), 5 mM MVA (turquoise), 10 mM MVA (grey), and 25 mM MVA (black). Cultivation conditions: Duplicates in M9 mineral medium with glucose (3 g/L), in shake flasks, at 30 °C, induced with 1 mM CHPA after 4 h

Since the introduction of the heterologous MVA pathway increased lycopene titres, we next tested whether precursor availability remained a bottleneck in the improved production background. For this purpose, *P. putida* CAP (pSEVA2312-Lyc-MVA), which exhibited the highest lycopene titres in the previous experiment, was cultivated with increasing concentrations of exogenously supplied mevalonate. As a central intermediate of the MVA pathway, mevalonate supplementation provides a direct strategy to enhance intracellular availability of IPP and DMAPP, thereby allowing evaluation of whether precursor supply still limits metabolic flux toward product formation. Cultures were supplemented with 1, 5, 10, or 25 mM mevalonate. In addition, two control conditions were included: *P. putida* CAP (pSEVA2312-Lyc-MVA) without mevalonate supplementation (0 mM) and *P. putida* CAP (pSEVA2312-Lyc) supplemented with 25 mM mevalonate (Fig. 5).

Growth curves, growth rates, and mCherry production rates remained comparable across all tested mevalonate concentrations in *P. putida* CAP (pSEVA2312-Lyc-MVA). Final OD_600_ values ranged from 1.4 to 1.7, while specific growth rates varied between 0.48 h⁻¹ and 0.52 h⁻¹. Likewise, mCherry production rates remained stable across all conditions (Figure S1).

In contrast, mevalonate supplementation affected lycopene accumulation after 24 h of cultivation (Fig. 5). The control strain *P. putida* CAP (pSEVA2312-Lyc), lacking the MVA pathway and therefore unable to metabolise the supplemented mevalonate, produced approximately 1 mg/L lycopene in the presence of 25 mM mevalonate. This titre was comparable to that observed without the addition of mevalonate. In comparison, cultures of *P. putida* CAP (pSEVA2312-Lyc-MVA) reached titres of up to 9 mg/L, depending on the concentration of supplemented mevalonate. The non-supplemented culture (0 mM) accumulated 6.22 ± 0.10 mg/L lycopene, which was comparable to the titres obtained with 1 mM and 25 mM mevalonate (approximately 6.7 mg/L). Supplementation with 5 mM or 10 mM mevalonate, however, increased lycopene titers to 9.47 ± 2.41 mg/L or 8.34 ± 1.69 mg/L, respectively.

**Fig. 6:**
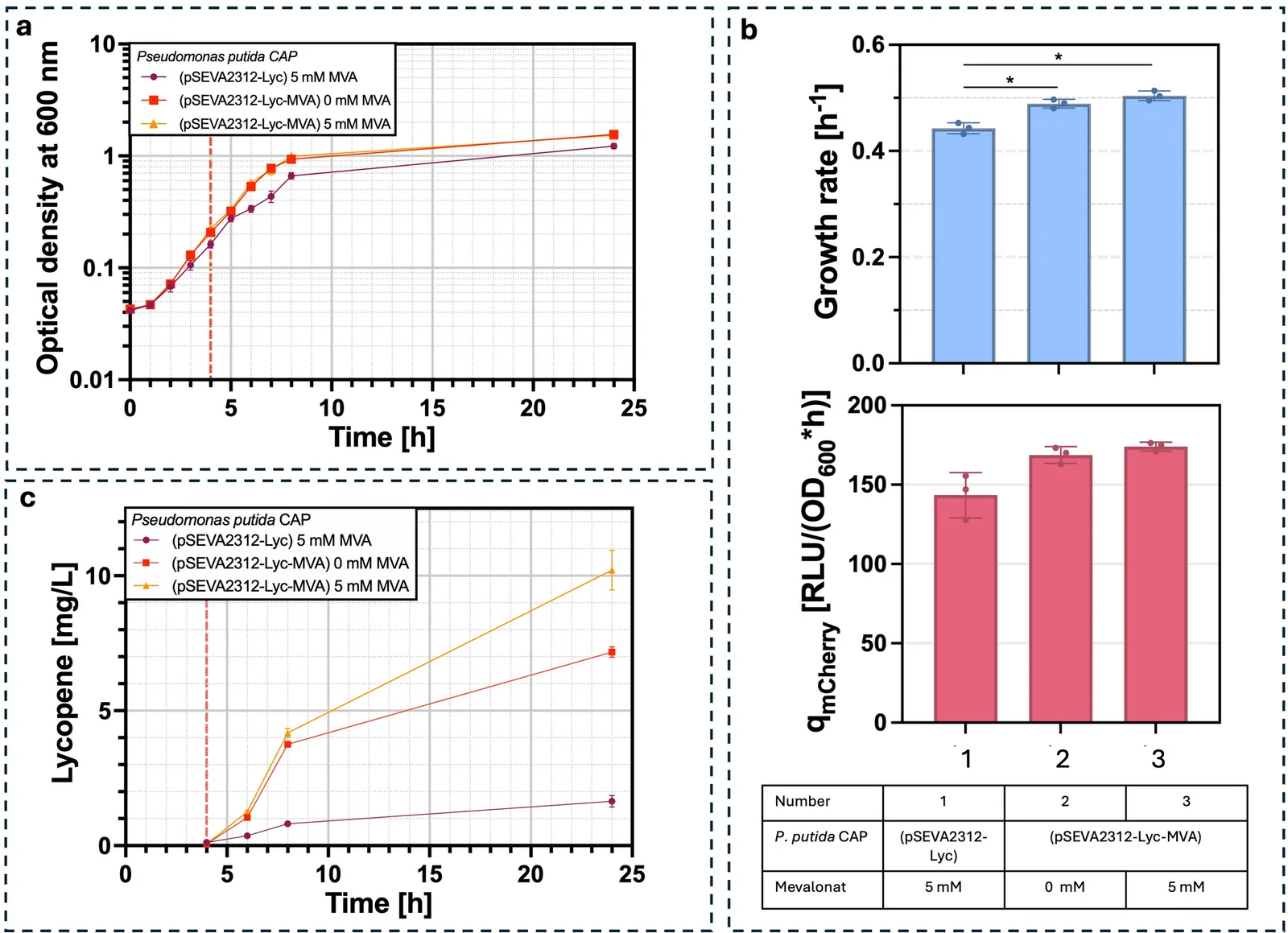
Growth (**a**), growth rate and capacity estimated from mCherry fluorescence rates (**b**), and lycopene production (**c**) of *P. putida* CAP carrying pSEVA2312-Lyc or pSEVA2312-Lyc-MVA supplemented with 5 mM MVA. Cultivation conditions: Triplicates in M9 mineral medium with glucose (3 g/L), shake flasks, 30 °C, induction with 1 mM CHPA after 4 h (dotted line). The exact time points used to calculate the growth rate and the mCherry production rate are provided in the supplements. The asterisks represent a statistical difference (*p* < 0.05 (*) for an unpaired two-tailed t-test)

Based on these results, supplementation with 5 mM mevalonate was selected for further characterisation. To evaluate the effect of enhanced precursor availability in more detail, growth behaviour, cellular capacity, and lycopene production were analysed under inducing conditions in *P. putida* CAP (pSEVA2312-Lyc-MVA) in the presence and absence of 5 mM mevalonate. As a control, *P. putida* CAP (pSEVA2312-Lyc), lacking the MVA pathway, was cultivated under identical conditions (Fig. 6).

All strains exhibited similar growth profiles during the early cultivation phase (Fig. 6a). The control strain lacking the heterologous MVA pathway showed a slightly lower growth rate (0.45 ± 0.01 h^− 1^), whereas strains harbouring the MVA pathway reached growth rates of around 0.5 h^− 1^ irrespective of mevalonate supplementation (Fig. 6b). Likewise, mCherry production rates remained comparable across all tested conditions (Fig. 6b).

After 24 h of cultivation, *P. putida* CAP (pSEVA2312-Lyc-MVA) supplemented with 5 mM mevalonate reached the highest lycopene titre (10.07 ± 0.74 mg/L), followed by the same strain cultivated without the addition of mevalonate (7.14 ± 0.19 mg/L). In contrast, the control strain lacking the MVA pathway accumulated only 1.64 ± 0.21 mg/L of lycopene, corresponding to an approximately six-fold lower titre than the mevalonate-supplemented strain carrying the MVA pathway (Fig. 6c).

Taken together, supplementation with 5 mM mevalonate further enhanced lycopene production in the MVA pathway harbouring strain *P. putida* CAP (pSEVA2312-Lyc-MVA), while growth behaviour and mCherry production remained largely unaffected across all tested conditions. This shows that lycopene production remained constrained by precursor availability even after the introduction of the heterologous MVA pathway.

### Bioreactor cultivation increases lycopene production

Because precursor engineering and mevalonate supplementation substantially increased lycopene titres but did not fully eliminate production constraints, we next asked whether process conditions become increasingly important at higher production levels. Based on its tightly regulated inducible expression, low basal lycopene production under non-induced conditions, and consistently high lycopene titers, *P. putida* CAP (pSEVA2312-Lyc-MVA) was selected for evaluation under bioreactor conditions. The controlled process environment in the bioreactor, particularly the improved oxygen transfer and higher process stability, was expected to be beneficial for cellular energy and redox metabolism and, therefore, provided a suitable setting to test whether additional process-dependent constraints beyond precursor supply would become apparent during heterologous lycopene production.

To this end, *P. putida* CAP (pSEVA2312-Lyc-MVA) was cultivated in a 1 L stirred-tank bioreactor and lycopene production was evaluated.

**Fig. 7:**
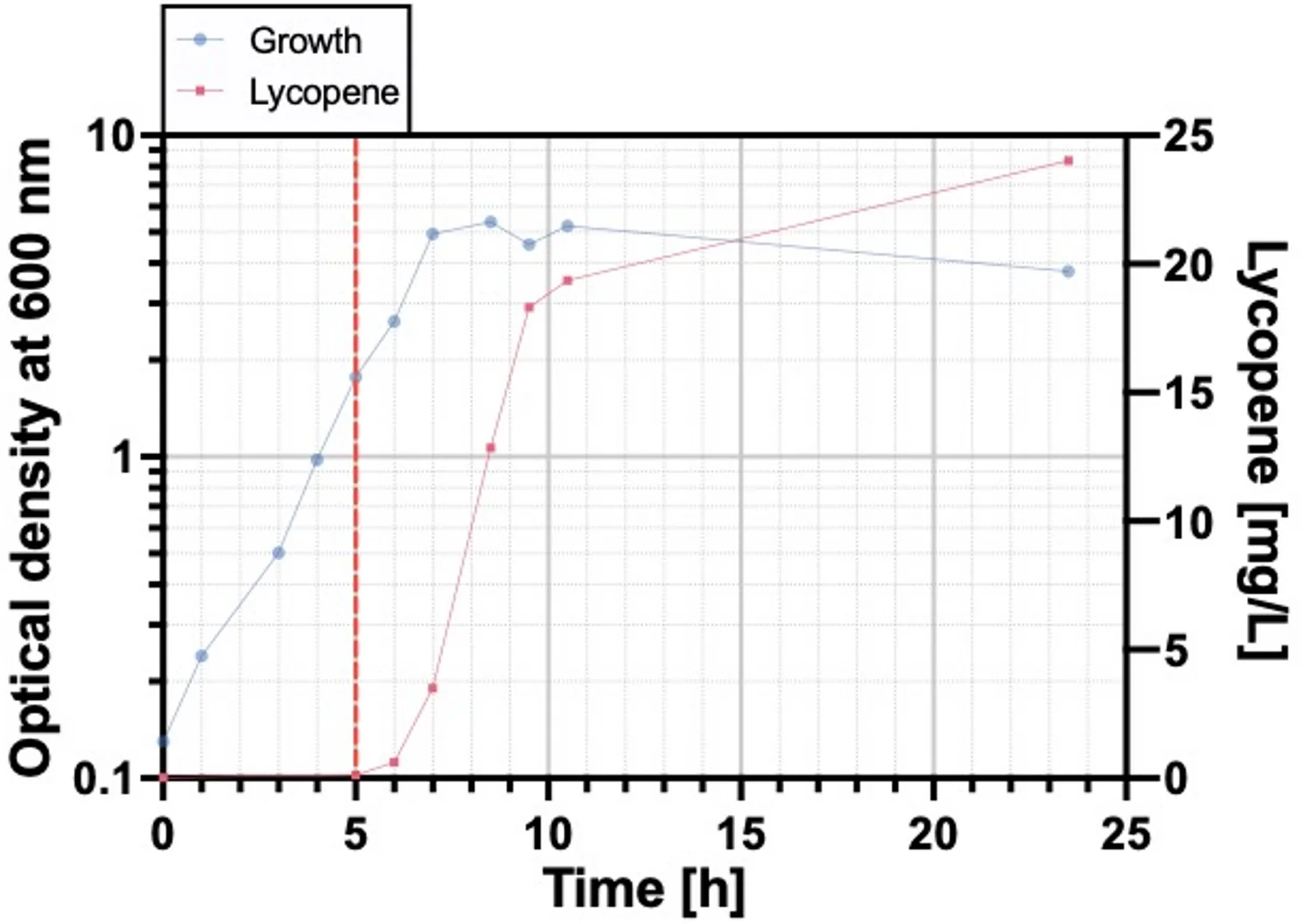
Growth (blue) and lycopene concentration (red) during batch cultivation of *P. putida* CAP (pSEVA2312-Lyc-MVA) in a stirred-tank bioreactor containing M9 mineral medium supplemented with 15 g/L glucose. The pH was maintained at 7, and pO₂ above 40%. The red dotted line marks the time point of induction with 1 mM CHPA

Figure 7 shows the growth and lycopene concentration throughout the bioreactor cultivation. Induction of the expression of the heterologous pathway occurred approximately 5 h after inoculation, as indicated by the red-dotted line. Lycopene production became detectable roughly one hour later, suggesting a short delay between activation of gene expression and product formation. After induction, the cells continued to grow for approximately two hours, reaching a maximal OD_600_ of around 5 before growth ceased. During the subsequent production phase, lycopene accumulation increased rapidly between 6 and 9 h of cultivation, reaching 18.31 mg/L after 9.5 h. Thereafter, production continued at a lower rate, resulting in a final lycopene concentration of 24.03 mg/L after 23.5 h of cultivation. During this phase, the OD_600_ slightly decreased from 5 to 4 toward the end of the process.

To evaluate whether nutrient limitation contributed to the abrupt cessation of growth, glucose concentrations were monitored during an independent bioreactor cultivation (Figure S2). Although this replicate exhibited a slightly lower initial OD, resulting in a reduced OD_600_ at the time of induction and a lower maximal biomass concentration, growth arrest occurred at the same time point relative to induction (Figure S3). Notably, glucose remained available in the medium after growth had ceased, indicating that carbon source depletion was not responsible for the observed growth limitation. The biomass-specific production, product-to-substrate yield (Y_P/S_) and biomass-specific production rate for both process runs are provided in the supplementary information (Table S2).

Compared to shake-flask cultivation, transfer to the 1 L bioreactor resulted in an approximately threefold increase in lycopene yield, highlighting the beneficial effect of controlled cultivation conditions on production performance. The improved performance under bioreactor conditions is consistent with a contribution of process-dependent energy and redox constraints, although these factors were not directly quantified in this study.

## Discussion

Efficient heterologous production of terpenoids in microbial hosts is often constrained by three principal factors: (i) cellular gene expression capacity, (ii) precursor availability and metabolic flux distribution, and (iii) cellular energy and redox metabolism. In this study, these constraint layers were systematically dissected in *P. putida* using lycopene production as a model system. The results allow a clearer hierarchy of constraints to be established: general gene expression capacity was not measurably limiting under the tested conditions, precursor availability emerged as the dominant bottleneck, and process-dependent physiological effects became more apparent at higher production levels.

Across all tested strains and conditions, mCherry production rates, used as a proxy for cellular capacity, remained largely unchanged despite pronounced differences in growth rates and lycopene accumulation. This decoupling between cellular capacity and growth strongly indicates that the observed phenotypes are not driven by limitations in ribosome availability or general gene expression resources. Instead, differences in promoter architecture revealed that regulatory characteristics of the expression system play a critical role in pathway performance.

The XylS/P_*m*_ system exhibited substantial basal activity, as indicated by similar expression levels under inducing and non-inducing conditions, whereas the CprK1/P_*DB3*_ system showed tight induction-dependent control with low basal expression (Figs. 3c and 4c). The similar lycopene titres observed under inducing and non-inducing conditions for the XylS/P_*m*_-driven construct suggest that even low basal expression levels are sufficient to produce enzyme concentrations that support lycopene biosynthesis. As a result, induction did not further enhance production and may instead have increased pathway imbalance and metabolic burden [41, 42]. This regulatory behaviour may therefore account, at least in part, for the reduced performance of the pSEVA438-based construct. Such an interpretation is supported by the known properties of the XylS/P_*m*_ promoter, which originates from the *P. putida* TOL plasmid pWW0 and is widely used as an inducible expression system because of its dose-dependent characteristics. At the same time, it is also known to exhibit considerable basal activity in its native host, likely owing to low constitutive activity of the XylS activator and the high compatibility of the regulatory architecture with the host’s transcriptional machinery [43, 44], so that target gene expression can occur even in the absence of an external inducer.

In contrast, the CprK1/P_*DB3*_ promoter present in the pSEVA2312 vector obviously provides tighter regulatory control. This more stringent regulation is consistent with the heterologous origin of the CprK1/P_*DB3*_ system, which is based on the CprK1 transcription factor from *D. hafniense* and therefore lacks any native activation signals in *P. putida* [45–47]. As a result, basal activity remains low in the absence of the specific inducer, enabling a clearer separation between inducing and non-inducing states. The CprK/P_*DB3*_ module has previously been implemented as an inducible expression system within the SEVA framework and related synthetic biology toolkits [48, 49], demonstrating reliable, tightly controlled gene expression. Such regulatory properties may facilitate improved pathway balancing and metabolic robustness during heterologous pathway expression in *P. putida*. These results indicate that the regulatory characteristics of the expression system have an influence on pathway performance.

To test whether precursor availability limits lycopene synthesis in *P. putida* CAP, a heterologous MVA pathway was subsequently introduced into both plasmid constructs. The resulting response clearly depended on the regulatory context of the expression system. While the introduction of the MVA operon into the pSEVA438 background resulted in reduced growth rates and early growth arrest upon induction, cells carrying the pSEVA2312-based construct showed the opposite trend and even displayed higher growth rates than the corresponding strain carrying only the lycopene operon. Although this increase may initially appear counterintuitive, the physiological effect of a heterologous MVA pathway is strongly condition dependent. In the lycopene-producing strain, lycopene biosynthesis creates a high demand for the isoprenoid precursors IPP and DMAPP, leading to precursor limitation and an associated metabolic imbalance. The heterologous MVA pathway appears to alleviate this bottleneck by supplementing the native MEP-derived precursor supply [50, 51]. Consequently, the reduction in physiological burden caused by precursor limitation seems to outweigh the additional burden associated with expressing the heterologous MVA pathway, resulting in the improved growth observed for *P. putida* (pSEVA2312-Lyc-MVA). This interpretation is consistent with previous studies showing that balanced MVA pathway expression can improve both carotenoid production and cell growth [52]. At the same time, the introduction of the heterologous MVA pathway caused a substantial increase in lycopene production, demonstrating that precursor supply is a major limiting factor for terpenoid biosynthesis in *P. putida* CAP. This effect was particularly evident in the pSEVA2312-based system, where implementation of the MVA pathway led to an almost 7.5-fold increase in lycopene titres compared with the corresponding strain carrying only the lycopene operon (Fig. 4c). Thus, vector architecture shaped the magnitude of the response, whereas the biological conclusion remained unchanged: enhanced precursor availability can markedly improve production performance when pathway expression is properly balanced. These findings are consistent with previous studies reporting that precursor availability at the level of IPP and DMAPP represents a major bottleneck in microbial terpenoid biosynthesis [25, 28, 53]. Introduction of heterologous MVA pathways has repeatedly been shown to enhance isoprenoid production by increasing intracellular precursor supply [25, 27]. Our results extend these observations to a plasmid-based expression system in *P. putida* and demonstrate that complementing the native MEP pathway can substantially improve production performance in this host.

Experiments involving mevalonate supplementation provided further support for precursor limitation. Several studies have shown that the addition of mevalonate to the growth medium can significantly increase isoprenoid production by raising the intracellular availability of the precursors IPP and DMAPP. For example, lycopene production in *E. coli* has been reported to increase in a dose-dependent manner upon mevalonate addition and to reach a plateau at higher concentrations [27, 28, 54]. A direct comparison of this study with *E. coli* production systems remains difficult, as these studies often employ different heterologous pathway architectures, additional metabolic engineering strategies, substantially higher biomass concentrations, and different cultivation conditions. Nevertheless, one study reported lycopene titres of approximately 200 mg/L in an *E. coli* strain supplemented with mevalonate (25 mM) with a total OD_600_ of around 3, highlighting the strong impact of precursor supplementation on terpenoid production performance [54].

The mevalonate feeding experiments revealed that supplementation with 5 mM mevalonate yielded the highest lycopene titres, whereas higher concentrations did not further increase production, suggesting pathway saturation (Fig. 5). This increase in lycopene production upon precursor supplementation further supports the conclusion that the intracellular availability of isoprenoid precursors represents a key limiting factor in the system. At the same time, the saturation observed at higher concentrations suggests that once precursor supply exceeds the catalytic capacity or balance of downstream pathway steps, further increases in precursor availability no longer translate into higher product formation. Consistent with this interpretation, optimisation of the downstream MVA pathway in *E. coli* by tuning the expression of mevalonate kinase (MVK), phosphomevalonate kinase (PMK), mevalonate diphosphate decarboxylase (MVD), and isopentenyl diphosphate isomerase (IDI) markedly increased metabolic flux and resulted in a lycopene yield of 219.7 mg/g DCW in shake-flask cultures [51]. This finding further underscores the importance of balanced precursor delivery rather than the mere introduction of an additional pathway. Although the results obtained in *E. coli* cannot be directly transferred to *P. putida*, systematic balancing of the heterologous MVA pathway could represent a promising strategy for further improving lycopene production in *P. putida*. The concentration-dependent response followed by saturation observed in our study is also consistent with the pattern previously reported by Martin et al. [27]. Considering the comparatively low biomass concentrations achieved in this study, it is likely that further process optimisation and cultivation to higher cell densities could enable substantially higher lycopene production in *P. putida* CAP (pSEVA2312-Lyc-MVA).

Scaling up the process under controlled bioreactor conditions resulted in a substantial increase in lycopene yield, demonstrating that improved process control can enhance production performance. Following induction, growth rapidly came to a halt, while lycopene production continued, suggesting a decoupling of biomass formation and product synthesis. The persistence of a glucose surplus throughout the cultivation rules out carbon limitation as the primary cause of the growth arrest, and the reproducibility of this phenotype further supports a systemic metabolic origin. One plausible interpretation is that production under these conditions imposed additional demands on oxygen-dependent metabolism, energy turnover, and redox homeostasis, which might become more apparent at higher production levels [17, 55]. Alternatively, the growth arrest could be the result of a metabolic imbalance caused by carbon overload. Following induction, the cells shift from growth to product formation, requiring a redistribution of cellular resources. If carbon uptake exceeds the cells’ metabolic capacity, intermediates or cofactors may accumulate and disrupt metabolic homeostasis. Therefore, a fed-batch process combined with a lower initial glucose concentration could promote a more controlled product formation and potentially reduce metabolic overload.

In addition, the accumulation of hydrophobic carotenoids, such as lycopene, in cellular membranes has been shown to alter membrane properties, potentially affecting membrane fluidity, permeability, and associated cellular processes. Such membrane-associated effects may contribute to growth arrest without necessarily causing immediate cell death [25, 41]. Thus, lycopene production has often been observed to continue after growth has ceased, indicating that product formation can persist under non-growing or maintenance conditions [17, 54]. Together, these observations suggest that the transition into the stationary phase after induction of gene expression is likely driven by a combination of metabolic stress and membrane-associated effects arising from active lycopene biosynthesis rather than by direct lycopene toxicity alone.

## Conclusion

Overall, this study demonstrates that metabolic burden during heterologous lycopene production in *P. putida* is governed by distinct constraint layers rather than by a single limiting factor. First, cellular resources required for transcription and translation were not limiting, as indicated by stable expression of the capacity reporter gene across all tested conditions. Instead, pathway performance was strongly influenced by the regulatory characteristics of the expression system, particularly basal activity and controllability.

Second, and most importantly, precursor availability and pathway flux represented the dominant constraint. Enhancing precursor supply through the introduction of a heterologous MVA pathway and targeted supplementation substantially improved lycopene production, while saturation behaviour at higher precursor levels indicated that the downstream enzyme capacity or pathway balance became limiting. Third, process conditions further modulated production performance. The higher titres achieved in the bioreactor show that controlled cultivation can alleviate additional process-dependent constraints, which may include effects on energy metabolism and redox balance. However, the specific contributions were not directly measured here and therefore remain an indirect interpretation.

Collectively, these findings demonstrate that efficient terpenoid production in *P. putida* requires coordinated optimisation of pathway regulation, precursor supply, and metabolic balance. The capacity-monitoring approach applied in this study provides a systematic framework for distinguishing these constraint layers and guiding future strain and process engineering strategies for isoprenoid-derived compounds.

## Supplementary Information

Below is the link to the electronic supplementary material.

Supplementary Material 1.

## Data Availability

All data supporting the findings of this study are available within the paper and its supplementary information.
