# Supplementary material for: Characterisation of metabolic burden in Pseudomonas putida reveals precursor limitation in heterologous lycopene production
Source: Microb Cell Fact. 2026 Sep 2;25:179. doi: 10.1186/s12934-026-03108-5 (PMC13540941; doi:10.1186/s12934-026-03108-5)
Supplement: Supplementary file 1 [file 12934_2026_3108_MOESM1_ESM.pdf]

Supplementary Information

for

Characterisation of metabolic burden in *Pseudomonas putida* reveals precursor limitation in heterologous lycopene production

by

Carina Meiners, Lucas Hermann, Mishela Stoja, Andreas Kremling, Katharina Pflüger-Grau*

Professorship of Systems Biotechnology, Technical University of Munich, Boltzmannstraße 15, 85748 Garching

***For correspondence:** Katharina Pflüger-Grau, Professorship of Systems Biotechnology, Technical University of Munich, Boltzmannstraße 15, 85748 Garching (Germany), Tel: +49 89 289 15765, e-mail: k.pflueger-grau@tum.de

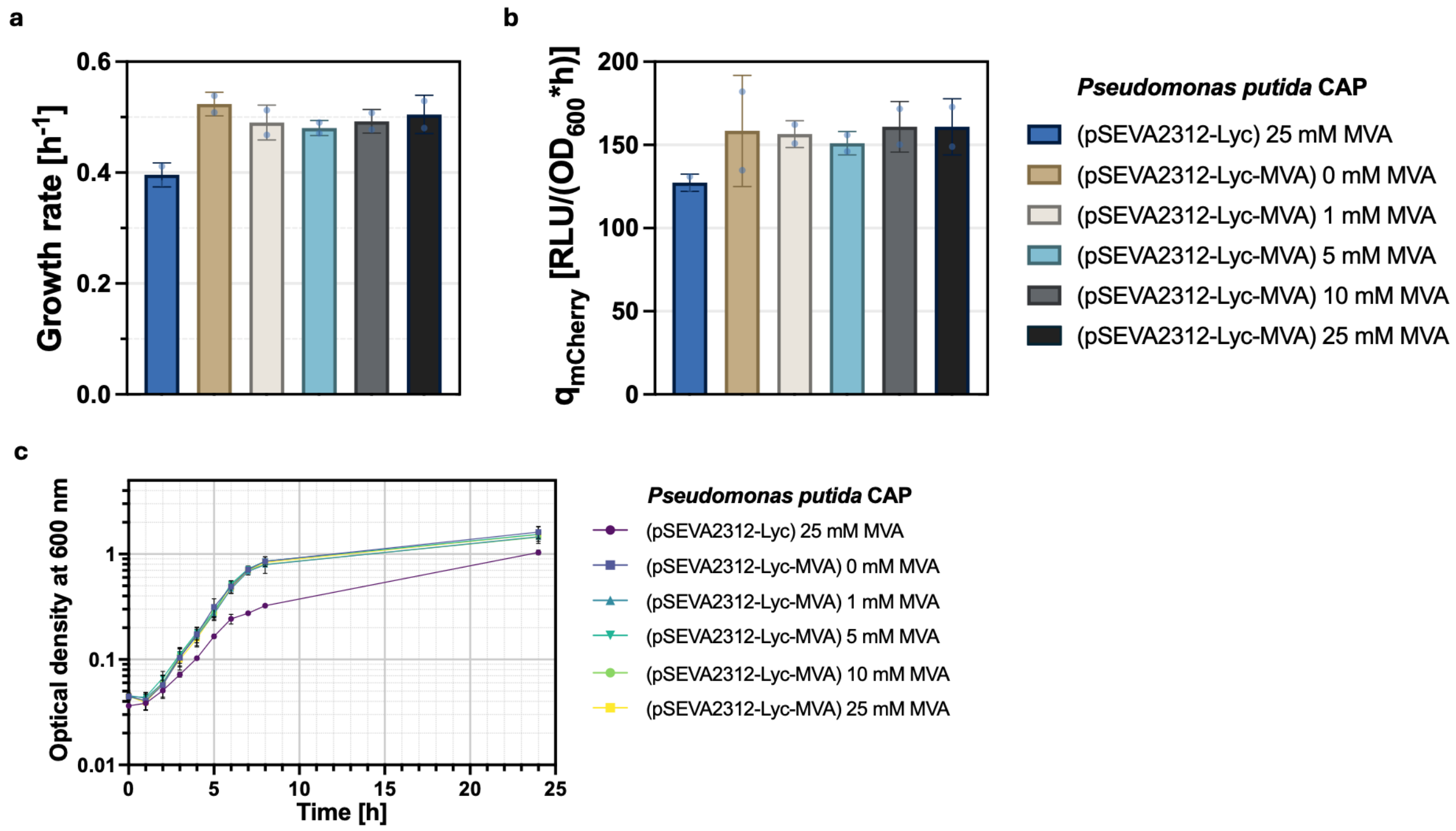

Figure S1 Growth rate (a), capacity estimated from mCherry fluorescence rate (b), and growth (c) of *P. putida* CAP (pSEVA2312-Lyc) and *P. putida* CAP (pSEVA2312-Lyc-MVA) supplemented with different concentrations of mevalonate.

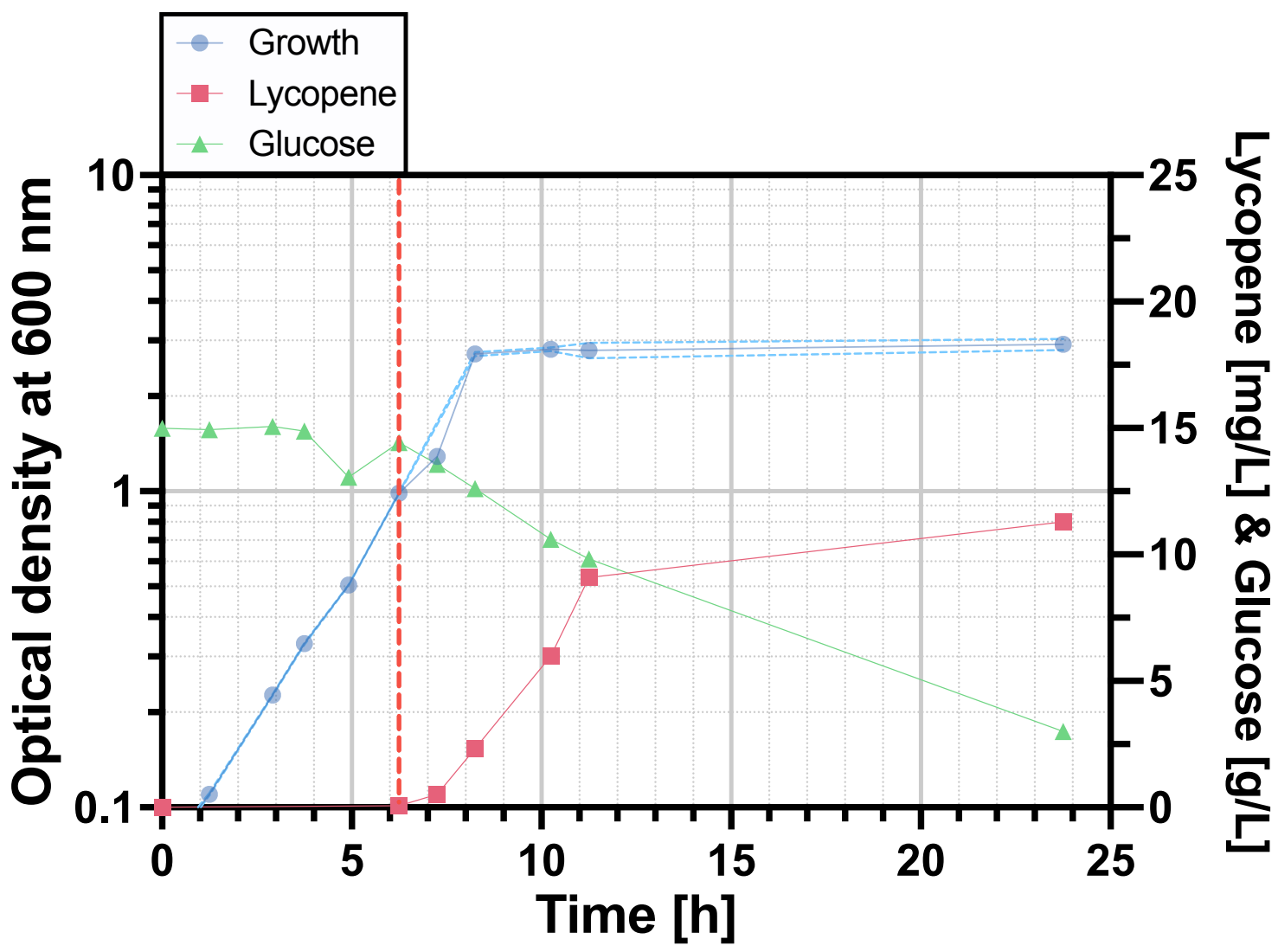

Figure S2 Growth (blue), lycopene concentration (red), and glucose concentration (green) during the repeated batch cultivation of *P. putida* CAP (pSEVA2312-Lyc-MVA) in a stirred-tank bioreactor containing M9 mineral medium supplemented with 15 g/L glucose. The pH was maintained at 7, and pO_2 above 40%. The red-dotted line marks the time point of induction with 1 mM CHPA.

a

b

c

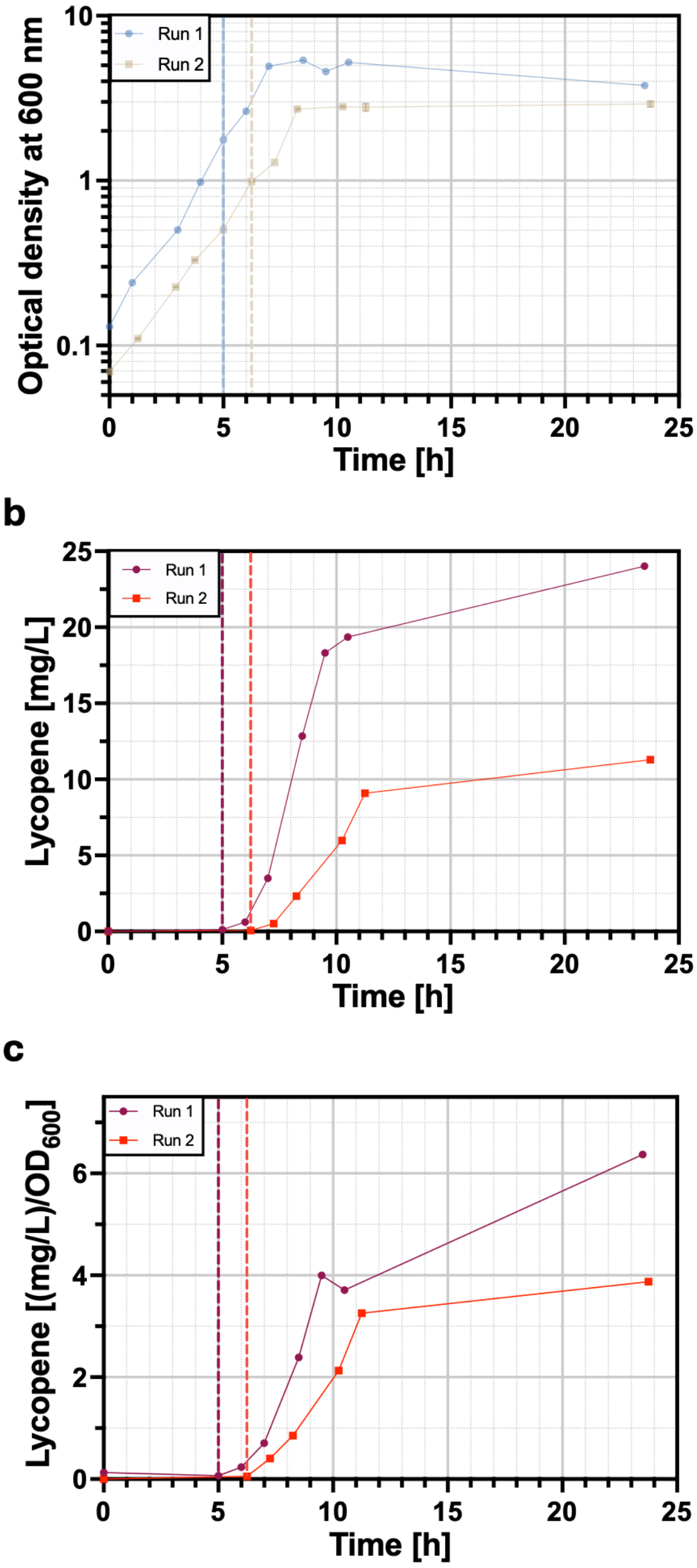

Figure S3 Growth (a) lycopene titer (b) and lycopene concentration normalized to the optical density (c) of *P. putida* CAP (pSEVA2312-Lyc-MVA) of two independent batch processes (Run 1 and Run 2) in a stirred-tank bioreactor. Batch processes were performed in M9 mineral medium supplemented with 15 g/L glucose. The pH was maintained at 7, and pO_2 above 40%. The dotted lines mark the time point of induction with 1 mM CHPA.

Table S1 Time intervals used for the determination of specific growth rates (μ) by linear regression, listed for each strain and experimental condition, and grouped according to the corresponding figures.

Strain		Time intervals for growth rate [h]
**Figure 2**		
P. putida CAP	(pSEVA2312-Lyc)	3-9
	(pSEVA2312-Lyc) not induced	3-9
	(pSEVA438-Lyc)	3-8
	(pSEVA438-Lyc) not induced	2-9
**Figure 3**		
P. putida CAP	(pSEVA2312-Lyc-MVA)	1-5
	(pSEVA2312-Lyc-MVA) not induecd	1-6
	(pSEVA438-Lyc-MVA)	1-6
	(pSEVA438-Lyc-MVA) not induced	0-7
**Figure 5**		
P. putida CAP	(pSEVA2312-Lyc) 5 mM MVA	1-5
	(pSEVA2312-Lyc-MVA) 0 mM MVA	1-6
	(pSEVA2312-Lyc-MVA) 5 mM MVA	1-6
**Figure S1**		
P. putida CAP	(pSEVA2312-Lyc)	2-6
	(pSEVA2312-Lyc-MVA) 0 mM	1-6
	(pSEVA2312-Lyc-MVA) 1 mM	1-7
	(pSEVA2312-Lyc-MVA) 5 mM	1-7
	(pSEVA2312-Lyc-MVA) 10 mM	1-7
	(pSEVA2312-Lyc-MVA) 25 mM	1-7

Table S2 Biomass-specific production, product to substrate yield ($Y_{P/S}$) and biomass-specific production rate of two independent batch processes (Run 1 and Run 2) with *P. putida* CAP (pSEVA2312-Lyc-MVA). Since only the optical density was measured during these experiments and no biomass concentration was determined, the correlation CDW = 0.35*OD_{600} was used. This correlation had previously been established internally for the same strain based on independent experiments. Furthermore, as no substrate concentrations were determined for process Run 2, the product-to-substrate yield $Y_{P/S}$ could not be calculated for this run.

Process run	Biomass-specific production* [mg/g_{CDW}]	$Y_{P/S}$** [g/g]	r_p** [mmol/g_{CDW}*h]
**Run 1**	26.51	0.0198	0.0104
**Run 2**	18.21	n.a	0.0048

* The biomass-specific production was calculated using the final biomass and product concentrations.

** The product-to-substrate yield $Y_{P/S}$ and the biomass-specific production rate (r_p) were calculated using data from 8.25 to 11.25 h of process time for Run 1 and from 7.0 to 10.5 h for Run 2.
